# Sport supplementation in competitive swimmers: a systematic review with meta-analysis

**DOI:** 10.1080/15502783.2025.2486988

**Published:** 2025-04-09

**Authors:** Raúl Domínguez, Inmaculada López-León, Javier Moreno-Lara, Esteban Rico, Antonio J. Sánchez-Oliver, Ángela Sánchez-Gómez, Javier Pecci

**Affiliations:** a Universidad de Sevilla, Departamento de Motricidad Humana Rendimiento Deportivo, Sevilla, Spain; b University of Lavras, Studies Research Group in Neuromuscular Responses (GEPREN), Lavras, Brazil; c Universidad de Córdoba, Departamento de Enfermería Farmacología y Fisioterapia, Facultad de Medicina y Enfermería, Córdoba, España; d University of Seville, Department of Physical Education and Sport, Seville, Spain

**Keywords:** Sport nutrition, ergogenic aid, sports performance, ergogenic supplementation, aquatic sport

## Abstract

**Background:**

Competitive swimmers have a high prevalence of sports supplement (SS) consumption. However, only a few SS are scientifically proven to be safe, effective, and legal. Therefore, before incorporating supplements to enhance performance and health in competitive swimming, it is crucial to conduct an analysis and review to assess their effects. The objective of this study was to analyze the demonstrated effects of SS, as reported in published studies, on the swimming performance of competitive swimmers.

**Methods:**

Following PRISMA guidelines, a systematic search was conducted across six databases for the selection of studies included in this review. Studies that analyzed the effects of sports supplementation compared to placebo were included and subjected to meta-analysis.

**Results:**

This revision included 23 studies, 16 of them (69.6%) qualified as excellent and 7 (30.4%) as good at the methodological level based on the punctuation in the PEDro scale. The systematic review included 422 swimmers (61.8% male, 38.2% female), with distances assessed ranging from 50 m to 800 m, including studies employing interval procedures. Creatine showed a significant effect (ES = −0.46; 95% CIs = −0.75 to −0.17, *p* = 0.002; I^2^ = 11%) on swimming performance, while the rest of the analyzed supplements did not show significant effects (all *p* > 0.05).

**Conclusions:**

Creatine supplementation demonstrated ergogenic benefits for competitive swimmers, although the evidence supporting the use of this supplement is still limited. Sodium bicarbonate and β-alanine may enhance performance in distances with higher glycolytic demands, while caffeine is effective at dosages of 3–6 mg/kg administered 60 min before exercise. Further research is needed to confirm the potential ergogenic effects of other supplements, such as beetroot juice.

## 1. Introduction

Competitive swimming requires athletes to cover a predetermined distance as quickly as possible using specific techniques such as front crawl, breaststroke, backstroke, or butterfly [1]. Success depends on overcoming water resistance through a combination of strength, power, and technical proficiency. Official swimming events range from 50-m to 1500-m, with durations ranging from around 20 s to 15.5 min [2]. The energy systems engaged during competition vary based on the event’s length and specific demands. These systems include the high-energy phosphagen system, glycolytic metabolism, and oxidative phosphorylation pathways, which rely on carbohydrates, fats, or proteins for energy production [3].

Swimming, as an aquatic sport, poses unique challenges to athletes, requiring them to minimize drag forces while optimizing propulsive forces [4]. These factors are essential in determining the physiological and energetic demands of the sport, often taking precedence over the event’s duration. [2]. In addition, at a higher competitive level and, therefore, at a faster swimming speed, the hydrodynamic forces are more relevant; therefore, both technical and strength demands rise as performance improves [5,6].

The different swimming modalities performed in a pool pose substantial physical challenges for swimmers, both in competitions and training. These challenges vary depending on the swimming style and stroke employed, involving factors such as body size, muscle strength, glycolytic capacity, neuromuscular function, coordination, and cardiorespiratory endurance [2]. For instance, in a 400 m front crawl event, the oxidative energy contribution is 81%, compared to 15.3% in shorter events such as the 50 m and 100 m front crawl [3].

Elite swimmers typically engage in rigorous, high-intensity, and high-volume training programs designed to meet the specific demands of their events, whether they are sprint, middle-distance, or distance races [7,8]. A retrospective observational study spanning 20 years revealed that peak performance was achieved through progressive increases in training load, with macrocycles lasting approximately 14–15 weeks, and significant training volume at intensities below 4 mmol⋅L^−1^ and above 6 mmol⋅L^−1^ [7]. These demanding programs involve double daily training sessions, dryland training focusing on strength and power, and interval training with varying recovery durations. The extensive preparation is designed to improve the athletes’ technical efficiency and prepare them for sustained high-level performances in competitive events [4–6].

In high-level sports, the competition is so closely matched that even small differences can significantly impact an athlete’s chances of winning [9]. A performance variation of just 1.6% during the Olympics can be the deciding factor between finishing first or fourth [10]. For this reason, many athletes, particularly those at the elite level, use sports supplements (SS) to optimize their performance [11,12]. Elite athletes use supplements for a variety of reasons, including enhancing power, preventing nutritional deficiencies, maintaining good health, reducing the risk of injuries, and improving sports performance [12]. Competitive swimmers frequently rely on nutritional support and SS to enhance physical performance and support overall health. The choice of SS often varies based on the swimmer’s discipline and race distance [3]. The consumption of SS is influenced by factors such as sex and competition level, with men and highly competitive athletes showing higher usage rates [13]. In fact, swimmers have been shown to have a higher prevalence of SS consumption compared to athletes in other sports, ranking among the top four sports for SS use during the 2000 Sydney Olympic Games [14,15].

However, only a limited number of dietary supplements have been scientifically proven to enhance sports performance, including caffeine, creatine, acid-base balance regulators (β-alanine and sodium bicarbonate, NaHCO₃), and beetroot juice (BRJ)/nitrate [12]. The effectiveness of these supplements is specific to the sport and the type of effort required [16]. Therefore, before incorporating supplements to enhance performance and health in competitive swimming, it is essential to analyze and evaluate their safety, effectiveness, and legality. To address this, the present systematic review aims to fill a gap in the literature by examining the effects of various nutritional supplements on swim-related performance in competitive swimmers, as no previous study has comprehensively assessed the ergogenic potential of SS in this population.

## 2. Materials and methods

### 2.1. Protocol

This systematic review was reported attending to the Preferred Reporting Items for Systematic review and Meta-Analyses (PRISMA) guidelines [17]. The PICOS model [18] was used for the definition of the inclusion criteria (see Table 1).

**Table 1.** PICOS criteria stablished in the present systematic review.

Parameter	Criteria
Population	Competitive swimmers
Intervention	Sport supplement for performance
Comparators	Placebo
Outcome	Time trial tests in swimming pool
Setting	Double-blind/single-blind and randomized cross-over design

### 2.2. Search strategy

A systematic search using keywords combined with Boolean operators was conducted across multiple databases for the selection of studies included in this review. The databases used were Dialnet, Directory of Open Access Journals, PubMed, SciELO, Scopus, and SportDiscus. The search strategy employed was as follows: (supplement* OR “ergogenic aid”) AND (swimming OR swimmer OR “aquatic sport”). The search date was restricted between 2000 and February 1 2023.

### 2.3. Eligibility criteria

Two researchers (J.M.-L. and E.R.) independently screened the search results and assessed study eligibility. Discrepancies were resolved through evaluation by a third researcher (I.L.-L.). Based on the PICOS criteria, the following inclusion criteria were established:

− Participants: competitive swimmers in swimming pool.
− Intervention: studies including sport supplementation used for enhancing sport performance.
− Comparison: placebo condition.
− Outcomes: studies including a time trial test in swimming pool.
− Setting: well-controlled articles including double-/single-blind and randomized cross-over design.

Exclusion criteria were:

− Language: studies published in a language different to English, Spanish, Portuguese or Italian.
− Studies not related to the topic of the present systematic review.
− Studies with a sample that does not include competitive swimmers in swimming pool.
− Studies focused on biochemical variables, body composition, training characteristics or any other parameter that is not specific swimming performance in swimming pool (time trial test).
− Studies which are not controlled with a placebo condition.

### 2.4. Data extraction

Two researchers (I.L.-L. and E.R.) independently reviewed each study included in the systematic review. Using a predefined data sheet, they extracted the following information from each study: sample details (size and participant characteristics), intervention (supplementation protocol), test used, and results. For studies employing a chronic supplementation protocol, results were calculated as the difference between experimental conditions, expressed as the percentage change (%) from pre- to post-intervention using the following formula: (medium value with supplementation – medium value with control condition)/medium value with control condition × 100. A third researcher (J.M.-L.) compared the two data sheets and resolved any discrepancies in the extracted information. For studies that reported the magnitude of the differences between the supplementation and placebo conditions, these differences were included. For studies that met the inclusion criteria but lacked sufficient data to process the results, a researcher (I.L.-L. and/or J.M.-L.) contacted the authors to request the missing data. Studies that failed to provide the required data and did not include sufficient results in the manuscript were excluded from the systematic review.

### 2.5. Quality assessment of studies

The methodological quality of the studies included in the systematic review was assessed using the PEDro scale, which consists of 11 items and has been validated [19]. Each item was scored with a “yes” (1 point) if the criterion was met or a “no” (0 points) if it was not. The maximum possible score for a study was 10, as the first item of the PEDro scale is not included in the final score. Based on the final scores, studies were classified as having excellent (9–10 points), good (6–8 points), fair (4–5 points), or poor (<3 points) methodological quality.

The quality assessment of each study was independently performed by two authors (J.M.-L. and I.L.-L.). Any discrepancies were discussed and resolved by consensus between the two researchers. If no agreement could be reached, a third author (E.R.) provided their opinion to finalize the evaluation.

### 2.6. Risk of bias assessment

The revised Cochrane risk-of-bias tool for randomized trials (RoB2) was employed to assess the potential bias in each study [19]. Using this assessment tool, the risk of bias was categorized as “low,” “some concern,” or “high risk” based on the evaluation of five primary domains of bias. The assessment of risk of bias within the studies was independently conducted by two authors (J.P. and R.D.). Any discrepancies identified during the review process were resolved through a consensus-seeking approach, with a third reviewer (A.S-O.) intervening to settle disagreements.

### 2.7. Meta-analyses

Although meta-analyses could be performed with two studies at least [20], meta-analyses were only performed if ≥3 studies were available [21] due to the low number of participants usually involved in sport sciences [22]. Effect sizes (ES, i.e. Hedges g) with 95% confidence interval were calculated extracting means and standard deviations from pre and post values for each outcome (i.e. time to complete the trial) in the intervention (i.e. supplementation) and comparator group (i.e. placebo group). A randomized effects model was employed for analyses. Data were standardized using post-intervention standard deviation values. ES for each comparison were interpreted using the following scale: <0.2 trivial, 0.2–0.6 small, >0.6–1.2 moderate, >1.2–2.0 large, >2.0–4.0 very large, >4.0 extremely large [23]. When multiple comparisons (e.g. data presented in different sets and not in total time) are presented in a single study and involving the same subjects in the same temporal frame, the sample size in the control (or intervention) group was proportionately divided to facilitate comparisons across multiple groups (Higgins et al., 2008). Because β-alanine and sodium bicarbonate (SB) presents a same mechanism of action (pH regulation) and these supplements have been analyzed combined in previous studies [24,25], it was assessed in the meta-analysis both SS combined. Heterogeneity was assessed using I^2^ statistics and classified as follows: <25% low, 25–75% moderate, >75% high [26]. Risk of publication bias was assessed by plotting the effect size (i.e. standardized mean differences) against the standard error for each study and each comparison. Meta-analyses were performed with Review Manager V.5.4.1 software (Cochrane Collaboration, Copenhagen, Denmark). Statistical significance was set at *p* < 0.05.

### 2.8. Certainty of evidence

Two assessors (J.P. and R.D.) appraised the level of evidence certainty, categorized as “high,” “moderate,” “low,” or “very low,” employing the Grading of Recommendations, Assessment, Development, and Evaluation (GRADE) framework [27]. The initial level of evidence for each outcome commenced at a high degree of certainty. However, this level was subsequently adjusted based on the following criteria: i) Risk of Bias in Studies: Assessments were downgraded by one level if a high risk of bias was detected in the included studies; ii) Indirectness: A low risk of indirectness was presumed as the default condition due to the precision of populations, interventions, comparators, and outcomes being ensured by the eligibility criteria; iii) Risk of Publication Bias: Downgrades occurred by one level if there was suspicion of publication bias; iv) Inconsistency: Assessments were downgraded by one level when substantial statistical heterogeneity (I2 > 75%) was present; v) Imprecision: One level of downgrading was applied when the number of participants for continuous-data comparisons was fewer than 300 [28]. Due to the study design included in this meta-analysis (i.e. RCTs crossover design), the number of participants was considered the number of participants computed in the meta-analyses as the sum of the intervention and control (e.g. a study with 10 participants involved in two experimental conditions – supplementation and control – was assessed as 20 participants) to assess this outcome in GRADE. In comparisons where an inadequate number of trials were available for meta-analysis, the evidence was automatically categorized as having a very low degree of certainty. Consequently, for outcomes not included in the meta-analyses, the evidence’s certainty should be regarded as very low.

### 2.9. Sensitivity analyses

Sensitivity analyses were conducted omitting each single study in meta-analyses for checking if data from any study substantially alters (i.e. changing results from non-significant to significant and vice versa) the findings of the present systematic review with meta-analysis.

## 3. Results

### 3.1. Study selection

**Figure 1.**
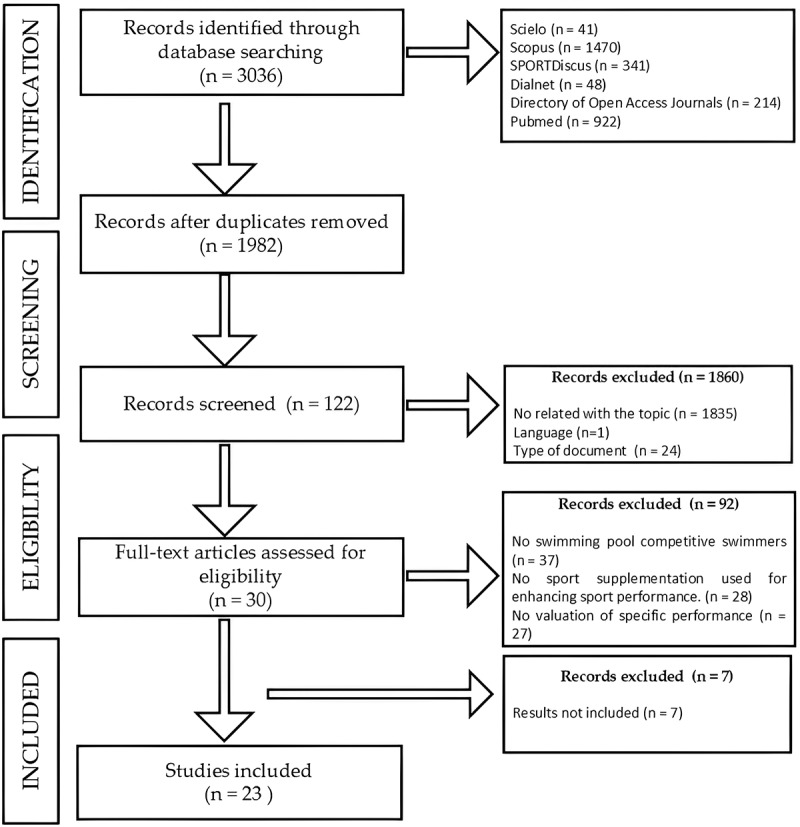
Selection of studies according to the PRISMA flowchart.

A total of 3,036 records were identified during the search, of which 1,054 were duplicates. Additionally, several studies were excluded for not being relevant to the study’s topic (*n* = 1,835), language (*n* = 1), or failing to meet the inclusion criteria regarding the type of study (*n* = 24). This left 92 documents as potentially eligible for the review. After applying the inclusion criteria, only 30 documents met the requirements for inclusion in the systematic review. However, seven studies were excluded due to the inability to obtain specific data on their results. Consequently, a total of 23 studies were selected for inclusion in this review (see flowchart in Figure 1).

### 3.2. Characteristics of the studies

The sample size of this systematic review includes a total of 422 swimmers, 261 (61.8%) of whom were males and 164 (38.2%) females. Of these, 7 studies exclusively included male swimmers, 3 focused solely on female swimmers, and 13 studies included swimmers of both sexes. Regarding the level of participants, 90 were categorized as trained swimmers, 38 as competitive swimmers, 35 as university non-elite, 60 as Division II athletes, and 20 as Division I athletes. Additionally, 84 were regional/national-level swimmers, and 95 were classified as international/elite swimmers.

In terms of SS analyzed, creatine (Cr) was the most studied supplement, appearing in 6 studies [29–34], followed by BRJ (4 studies) [4,35–37], β-alanine (3 studies) [38–40], caffeine (2 studies) [41,42], L-arginine/L-citrulline (2 studies) [43,44] and SB (2 studies) [45,46]. Other SS, such as probiotics [47] and rice germ [48], were each analyzed in a single study. Additionally, three studies evaluated the combined effect of co-ingesting SB with Cr [49], β-alanine [40] and caffeine [50].

Regarding the specific swimming test used, the distances are located in a range from 25-m [34] to 800-m [36]. The most analyzed distance was 100 m, reported in 9 studies [4,29,31,33,37,40,44,47,49], with three studies employing interval protocols ranging from 2 sets [33,49] to 6 sets [4]. Eight studies focused on 200 m [37,40,43,44,46,48,50], one of which used an interval protocol [50], followed by 50-m used in 7 studies [29,31,34,36,41,48], with two studies incorporating interval procedures [31,36]. Eight studies selected 200-m [37,40,43,44,46,48,50] with one of the studies using an interval procedure [50], followed by 50-m used in 7 studies [29,31,34,36,41,48], two of them using an interval procedure [31,36]. Other distances analyzed included 25 m [34,45], 168-m [35], 400-m [32,39], 500-m [47], 800-m [36], 50-yar [30,42], and 100-yar [30]. In addition, one study converted the differences in performance between pre- and post-supplementation protocol in different competition distances with logarithmic models to unify swimming performance [38].

### 3.3. Results of the studies are included in the systematic review

Table 2 shows the summary of the studies included in this systematic review.

**Table 2.** Summary of studies that have analyzed the effect of sport supplementation.

Supplement	Reference	Participants	Experimental conditions	Supplementation protocol	Test	Outcomes	Results
Creatine	Vatani [27]	20 males trained swimmers	EG1: Cr (*n* = 10) EC2: PLAC (*n* = 10)	EG1: 4 × 5 g/day with apple juice along 6 days EG2: idem with PLAC	50-m of breastroke*	Total time (s)	↓ 4.52% EG1 (from 53.1 ± 3.7 to 50.7 ± 2.8; *p* = 0.04) ↓ 2.69% EG2 (from 53.44 ± 2.76 to 52 ± 3.78; *p* > 0.05)
					100-m of breastroke	Total time (s)	↓ 2.46 % EG1 (from 122 ± 12.4 to 119 ± 13.9; *p* > 0.05) ↓ 0.83 % EG2 (from 120 ± 10.3 to 119 ± 10.7; *p* > 0.05)
	Selsby [28]	30 division II (18 males and 12 females) (19.3 ± 0.2 years)	EG1: Cr (*n* = 10) EC2: PLAC (*n* = 10)	EG1: 300 mg/kg divided in 4 dosages along 5 days +2.25 g/day along 9 days EG2: idem with PLAC	50-yard*	Total time (s)	↓ 1.03% EG1 (from 26.18 ± 0.32 to 25.91 ± 0.34; *p* = 0.12) ↑ 1.00% EG2 (from 26.99 ± 0.71 to 27.26 ± 0.49; *p* > 0.05) EG1 increase performance compared to EG2 (*p* < 0.05)
					100-yard*	Total time (s)	↓ 1.87 % EG1 (from 58.20 ± 0.36 to 57.11 ± 0.33; *p* > 0.05) ↑ 0.33% EG2 (from 59.90 ± 0.99 to 60.10 ± 0.99; *p* > 0.05) EG1 increase performance compared to EG2 (*p* = 0.003)
	Mendes [29]	18 competitive swimmers (12 males and 6 females) (EG1: 19.4 ± 2.6 years; EG2: 19.8 ± 2.3 years)	EG1: Cr (*n* = 9) EC2: PLAC (*n* = 9)	EG1: 20 g/day divided in 4 dosages along 8 days EG2: idem with PLAC	50-m	Total time (s)	↑ 0.61% EG1 (from 29.62 ± 3.77 to 29.80 ± 3.92; *p* > 0.05) ↑ 1.05% EG2 (from 30.62 ± 3.94 to 30.94 ± 4.12; *p* > 0.05)
					100-m	Total time (s)	↑ 0.50% EG1 (from 69.47 ± 18.68 to 69.82 ± 18.89; *p* > 0.05) ↑ 1.35% EG2 (from 67.17 ± 8.40 to 68.08 ± 8.74; *p* > 0.05)
					3 × 3 x 50-m. Recovery: 0.5 and 2.5 min	Sum of each set (s)	1º Set 50-m: ↑ 1.02% EG1 (from 97.75 ± 12.04 to 98.75 ± 11.51; *p* > 0.05) ↑ 0.50% EG2 (from 101.20 ± 12.71 to 101.71 ± 13.29; *p* > 0.05) 2º Set 50-m: ↓ 0.03% EG1 (from 95.76 ± 10.79 to 95.73 ± 11.17; *p* > 0.05) ↑ 0.71% EG2 (from 99.71 ± 12.27 to 100.42 ± 13.01; *p* > 0.05) 3º Set 50-m: ↑ 0.75% EG1 (from 94.27 ± 10.04 to 94.98 ± 10.19; *p* > 0.05) ↑ 0.46% EG2 (from 99.14 ± 12.35 to 99.60 ± 13.43; *p* > 0.05)
	Juhász [31]	16 males international level (15.9 ± 1.6 years)	EG1: Cr (*n* = 8) EC2: PLAC (*n* = 8)	EG1: 20 g/day divided in 4 dosages along 5 days EG2: idem with PLAC	2 × 100-m. Recovery: 4 min*	Time of each set (s)	1º 100-m: ↓ 3.7% EG1 (from 50.69 ± 1.41 to 48.86 ± 1.34; *p* = 0.034) ↑ 0.54% EG2 (from 50.13 ± 1.25 to 50.40 ± 1.28; *p* > 0.05) 2º 100-m: ↓ 3.8% EG1 (from 50.39 ± 1.38 to 48.53 ± 1.35; *p* = 0.026) ↑ 0.26% EG2 (from 50.01 ± 1.16 to 50.14 ± 1.24; *p* > 0.05)
	Azizi [32]	20 female competitive swimmers (20.9 ± 1.6 years)	EG1: Cr (*n* = 10) EC2: PLAC (*n* = 10)	EG1: 20 g/day divided in 4 dosages along 6 days EG2: idem with PLAC	25-m	Total time (s)	↓ 2.82% EG1 (from 18.70 ± 0.72 to 18.18 ± 0.88; *p* > 0.05) ↑ 1.30% % EG2 (from 18.59 ± 2.27 to 18.83 ± 2.12; *p* > 0.05)
					50-m	Total time (s)	↓ 4.45% EG1 (from 46.1890 ± 3.398 to 44,13 ± 2.88; *p* > 0.05) ↑ 0.82 % EG2 (from 44.46 ± 5.01 to 44.83 ± 3.56; *p* > 0.05)
	Anomasiri [30]	38 males trained swimmers (20.07 years)	EG1: Cr (*n* = 19) EC2: PLAC (*n* = 19)	EG1: 10 g/day with 30 g orange juice powder along 7 days EG2: only 30 g orange juice powder	400-m*	Total time and the last 50-m (s)	Total time: ↓ 4.17% EG1 (from 413.12 ± 107.73 to 395.89 ± 91.13; *p* = 0.005) ↓ 1.98% EG2 (from 416.07 ± 104.95 to 407.83 ± 96.80; *p* = 0.044) Last 50-m: ↓ 3.07% EG1 (from 47.91 ± 12.25 to 46.44 ± 11.65; *p* = 0.001) ↓ 1.97% EG2 (from 49.23 ± 11.39 to 48.26 ± 11.00; *p* = 0.081)
Beetroot juice	Lowings [33]	10 regional level (5 males and 5 females) (20.0 ± 1.0 years)		EC1: 140 ml (12.5 mmol NO_3_^−^) (180 min before) EC2: idem with PLAC	168-m backstroke*	Total time and two halves (s)	Total time: ↓ 0.9% EC1 vs EC2 (130.37 ± 8.10 vs 131.59 ± 9.09; *p* = 0.144) First half: ↓ 0.5% EC1 vs EC2 (62.85 ± 4.03 vs 63.14 ± 5.50; *p* = 0.678) Second half: ↓ 1.4% EC1 vs EC2 (67.52 ± 4.33 vs 68.45 ± 3.88; *p* = 0.062)
	Pospieszna [34]	11 female university nonelite (20.9 ± 1.3 years)	EC1: BRJ EC2: carrot juice EC3: PLAC	EC1: 500 ml a combined BRJ and chokeberry juice (10.2 mmol NO_3_^−^) along 8 days (180 min before) EC2: baseline before EC1 EC3: 500 ml carrot juice (10.2 mmol NO_3_^−^) along 8 days (180 min before) EC4: baseline before EC3	6 × 50-m. Recovery: not specified*	Time of each set (s)	3º 50-m: ↓ 2.1% (36.43 ± 2.01 vs 37.19 ± 2.23; *p* < 0.05) 4º 50-m: ↓ 4.2 EC1 vs EC2 (36.20 ± 2.24 vs 37.72 ± 2.50; *p* < 0.01); ↓ 3.3% EC3 vs EC4 (36.42 ± 2.42 vs 37.59 ± 1.99; *p* < 0.05) 5º 50-m: ↓ 4.4% EC1 vs EC2 (36.45 ± 2.44 vs 38.05 ± 3.13; *p* < 0.01); ↓ 3.1% EC3 vs EC4 (36.57 ± 2.37 ± vs 37.70 ± 2.14; *p* < 0.05) 6º 50-m: ↓ 5.8% EC1 vs EC2 (36.06 ± 2.86 vs 38.15 ± 2.99; *p* < 0.01); ↓ 4.3% EC3 vs EC4 (36.20 ± 2.44 vs 37.75 ± 2.39; *p* < 0.05)
					800-m*	Total time (s)	↓ 1.1% EC1 vs EC2 (690.41 ± 50.16 vs 697.84 ± 50.56; *p* < 0.05) ↓ 2.1.% EC2 vs EC4 (681.64 ± 43.05 vs 696.38 ± 43.61; *p* < 0.001)
	Esen [35]	10 university nonelite (5 males and 5 females) (22 ± 6 years)	EC1: BRJ EC2: PLAC	EC1: 140 ml (8.0 mmol NO_3_^−^) along 3 days (180 min before) EC2: idem with PLAC	100-m	Total time (s)	↑ 0.1% EC1 vs EC2 (69.5 ± 7.1 vs 69.4 ± 7.3; *p* > 0.05)
					200-m	Total time (s)	↑ 0.1% EC1 vs EC2 (152.6 ± 14.1 vs 152.5 ± 14.1; *p* > 0.05)
	Moreno [4]	13 national level (7 males and 6 females) (males: 16.4 ± 1.4 years and females: 15.3 ± 1.8 years)	EC1: BRJ EC2: PLAC	EC1: 70 ml (6.4 mmol NO_3_^−^) (180 min before) EC2: idem with PLAC	6 × 1000-m. Recovery: 7 min	Time of each set (s)	1º 100-m: ↓0.58% EC1 vs EC2 (63.69 ± 4.11 vs 64.06 ± 5.04; *p* > 0.05) 2º 100-m: ↓ 0.13% EC1 vs EC2 (64.13 ± 4.85 vs 64.21 ± 4.85; *p* > 0.05) 3º 100-m: ↓ 0.49 EC1 vs EC2 (64.42 ± 5.23 vs 64.73 ± 4.65; *p* > 0.05) 4º 100-m: ↓ EC1 vs EC2 (64.50 ± 4.73 vs 30.85 ± 1.92; *p* > 0.05) 5º 100-m: ↓ 0.66 EC1 vs EC2 (65.20 ± 5.06 vs 65.64 ± 3.51; *p* > 0.05) 6º 100-m: ↓ 1.51% EC1 vs EC2 (64.77 ± 4.83 vs 65.76 ± 3.68; *p* > 0.05)
β-alanine	Chung [36]	32 elite and subelite swimmers (15 males and 17 females) (22.6 ± 2.8 years)	EG1: β-alanine (*n* = 19) EG2: PLAC (*n* = 13)	EG1: 4.8 g/day divided in 4 dosages along 4 weeks +3.2 g/day divided in 3 dosages along 8 weeks EG2: idem with PLAC	Log-transformed from competition	Total time (s)	0% EG1 (from 454.6 ± 52.5 to 454.6 ± 52.6; *p* > 0.05) ↓ 0.1% EG2 (from 479.0 ± 70.4 to 478.6 ± 70.7; *p* > 0.05) Not differences between GEs
	Norberto [37]	13 national level (8 males and 5 females) (males: 20.25 ± 1.98 years and females: 20.0 ± 2.92 years)	EG1: β-alanine EG2: PLAC	EG1: 4.8 g/day divided in 6 dosages along 6 weeks	400-m	Total time (s)	↑ 0.1% EG1 (from 288.9 ± 18.6 to 289.2 ± 21.0; *p* > 0.05) 0% EG2 (from 292.2 ± 22.6 to 292.1 ± 24.2; *p* > 0.05) Not differences between GEs
	Painelli [38] *(β-alanine Protocol)*	16 trained swimmers (12 males and 6 females) (EG1: 18.62 ± 1.99 years; EG2: 20.16 ± 5.91 years)	EG1: β-alanine (*n* = 9) EG2: PLAC (*n* = 7)	EG1: 3.2 g/day divided in 4 dosages along 1 week +6.4 g/day divided in 4 dosages along 4 weeks EG2: idem with PLAC	100-m*	Total time (s)	↓ 2.09% EG1 (from 62.18 ± 7.23 to 60.88 ± 7.42; *p* > 0.05) ↑ 0.42% EG2 (from 64.79 ± 8.52 to 65.06 ± 9.37; *p* > 0.05) EG1 different to EG2 in POST (*p* = 0.029)
					200-m*	Total time (s)	↓ 2.01% EG1 (from 139.1 ± 16.2 to 136.3 ± 16.1; *p* = 0.002) ↓ 0.07% EG2 (from 144.3 ± 18.2 to 144.2 ± 19.1; *p* > 0.05)
Sodium bicarbonate/citrate	Lindh [44]	9 males elite level (20.4 ± 1.7 years)	EC1: SB EC2: PLAC EC3: Control	EC1: 300 mg/kg in capsules (from 90 to 60 min before) EC2: 200 mg/kg in capsules (from 90 to 60 min before)	200-m*	Total time (min:s)	↓ 1.6% EC1 vs EC2 (1:52.2 ± 4.7 vs 1:54.0 ± 3.6; *p* = 0.04) ↓ 1.3% EC1 vs EC3 (1:52.2 ± 4.7 vs 1:53.7 ± 3.8; *p* = 0.03)
	Siegler [43]	14 university nonelite (6 males and 8 females) (age not specified)	EC1: SB EC2: PLAC	EC1: 300 mg/kg with 0.5 l water (150 min before) EC2: idem with PLAC	8 × 25-m. Recovery: 5 seconds*	Total time (s)	↓ 2.3% EC1 vs EC2 (159.4 ± 25.4 vs 163.2 ± 2 5.6; *p* = 0.04)
Caffeine	Lara [39]	14 males national level (20.2 ± 2.6 years)	EC1: CAFF EC2: PLAC	EC1: 3 mg/kg (60 min before) EC2: idem with PLAC	50-m in the habitual swimming style*	Total time (s)	↓ 1.2% EC1 vs EC2 (27.45 ± 3.21 vs 27.77 ± 3.43; *p* = 0.01)
	Vanata [40]	30 division II (18 males and 12 females) (19.5 ± 1.4 years)	EC1: CAFF EC2: PLAC	EC1: 3 mg/kg (30 min before) EC2: idem with PLAC	50-yard*	Total time (s)	↓ 0.9% EC1 vs EC2 ( = 27.27 ± 3.65 vs 27.51 ± 3.74; *p* = 0.009)
L-arginine/L-citrulline	Esen and Karayigit [41]	8 males regional level (25 ± 5 years)	EC1: L-arginine (*n* = 8) EC2: PLAC (*n* = 8) EC3: Control	EG1: 8 g/day along 8 days (90 min before) EG2: idem with PLAC	200-m	Total time and two halves (s)	Total time: ↓ 1.1% EC1 (146.02 ± 10.34) vs EC2 (147.58 ± 10.86; *p* > 0.05) and ↓ 2.3% EC3 (149.40 ± 9.88; *p* > 0.05) First half: ↓ 0.3% EC1 (71.01 ± 4.85) vs EC2 (71.25 ± 4.90; *p* > 0.05) and ↓ 71.8% EC3 (71.85 ± 5.08; *p* > 0.05) Second half: ↓ 1.7% EC1 (75.02 ± 5.75) vs EC2 (76.34 ± 6.00; *p* > 0.05) and ↓ 3.3% EC3 (77.55 ± 5.17; *p* > 0.05)
	Esen [42]	15 regional level (10 males and 5 females) (25 ± 7 years)	EG1: L-arginine (*n* = 5) EG2: L-citrulline (*n* = 5) EG3: PLAC (*n* = 5)	EG1: 8 g/day along 8 days (90 min before) EG2: idem with L-citrulline EG3: idem with PLAC	100-m	Total time (s)	↓ 1.47% EG1 (from 67.97 ± 06.91 to 66.97 ± 05.89; *p* > 0.05) ↓ 1.65% EG2 (from 70.35 ± 05.21 to 69.19 ± 04.40; *p* > 0.05) ↓ 1.28% EG3 (from 82.06 ± 12.55 to 81.01 ± 11.45; *p* > 0.05)
Probiotic (B. longum 35,624)	Carbuhn [45]	20 university females division I (age not specified)	EG1: SB B. longum 35,624 (*n* = 10) EC2: PLAC (*n* = 10)	EG1: 4 mg/day along 6 weeks EG2: idem with PLAC	100-m	Total time (s)	↑ 0.2% EG1 (from 61.2 ± 2.2 to 61.3 ± 2.2; *p* > 0.05) ↓ 1.6% EG2 (from 64.8 ± 2.2 to 63.8 ± 2.1; *p* > 0.05)
					500-m	Total time (s)	↓ 4.4% EG1 (from 447 ± 21.9 to 437 ± 18.6; *p* > 0.05) ↓ 3.1% EG2 (from 457 ± 23.9 to 443 ± 18.5; *p* > 0.05)
Rice germ	Rondanelli [46]	27 national and regional level (20 males and 7 females) (34.7 ± 7.5 years)	EG1: Rice germ (*n* = 14) EG2: PLAC (*n* = 14)	EG1: 2 × 25 g/day (breakfast and afternoon) along 5 weeks	50-m	Total time (s)	↑ 0.36% EG1 (from 30.46 ± 2.94 to 30.57 ± 3.02; *p* > 0.05) ↓ 0.09% EG2 (from 33.64 ± 4.17 to 33.61 ± 4.24; *p* > 0.05)
					200-m*	Total time (s)	↓ 1.85% EG1 (from 161.38 ± 19.29 to 158.39 ± 17.63; *p* < 0.05) ↓ 0.46% EG2 (from 178.85 ± 43.01 to 178.02 ± 42.96; *p* > 0.05)
Caffeine + sodium bicarbonate	Pruscino [48]	6 males elite level (age not specified)	EC1: CAFF EC2: SB EC3: CAFF + SB E4: PLAC	EC1: 300 mg/kg with divided in 7 dosages (from 120 to 30 min before) EC2: 6 mg/kg caffeine 45 min before exercise EC3: EC1 + EC2 EC4: idem EC3 with placebo	2 × 200-m. Recovery: 30 min	Total time of each 200-m (min:s)	1º 200-m: not differences between CEs (EC1: 2:02.42 ± 3.17; EC2: 2:03.01 ± 3.68; EC3: 2:01.69 ± 3.19; EC4: 2:03.77 ± 3.21; *p* = 0.06) 2º 200-m: not differences between CEs (EC1: 2:03.90 ± 3.58; EC2: 2:02.62 ± 4.16; EC3: 2:01.70 ± 2.84; EC4: 2:04.22 ± 3.75; *p* = 0.06) EC2 increase more the time for covering the 2º 100-m compared to EC1 (*p* < 0.01) and EC3 (*p* < 0.05)
Creatine + sodium bicarbonate	Mero [47]	16 national level (8 males and 8 females) (males: 18.7 ± 0.7 years and females: 17.3 ± 0.8 years)	EC1: Cr + SB 2: PLAC	EC1: 20 g/day of Cr divided in 4 dosages along 6 days +300 mg/kg SB in capsules (120 min before) CE2: idem with placebo	2 × 100-m. Recovery: 10 min	Total time (s)	1º 100-m: ↓ 0.2% EC1 vs EC2 (62.8 ± 1.2 vs 62.9 ± 1.0; *p* > 0.05) 2º 100-m: ↓ 1.6% EC1 vs EC2 (62.9 ± 1.2 vs 63.9 ± 1.0; *p* > 0.05) EG1 increase more the time for covering the 2º 100-m in EG2 compared to EG1 (*p* < 0.05)
β-alanine + sodium bicarbonate	Painelli [38] *(β-alanine + sodium bicarbonate Protocol)*	16 trained swimmers (12 males and 6 females) (EC1/EC2: 20.71 ± 3.35 years; EC3/EC4: 18.71 ± 2.75 years)	EC1: β-alanine (*n* = 9) EC2: β-alanine + SB (*n* = 9) EC3: SB EC4: PLAC (*n* = 7)	EC1: 3.2 g/day divided in 4 dosages along 1 week +6.4 g/day divided in 4 dosages along 4 weeks + PLAC EC2: idem EC1 + 300 mg/kg SB with 0.5 l water (90 min before) EC3: PLAC + + 300 mg/kg SB with 0.5 l water (90 min before) EC4: idem with PLAC	100-m*	Total time (s)	↓1.40% EC1 (from 60.01 ± 4.31 to 59.17 ± 4.67; *p* = 0.49) ↓ 2.80% EC2 (from 60.01 ± 4.31 to 58.33 ± 4.97; *p* = 0.022) ↓ 2.67% EC3 (from 63.25 ± 5.55 to 61.56 ± 5.05; *p* = 0.051) ↑ 1.08 % EC4 (from 63.25 ± 5.55 to 63.93 ± 6.02; *p* > 0.05)
					200-m*	Total time (s)	↓ 1.22% EC1 (from 131.6 ± 8.8 to 130.0 ± 8.6; *p* = 0.06) ↓ 2.13% EC2 (from 131.6 ± 8.8 to 128.8 ± 9.0; *p* < 0.001) ↓ 1.46% EC3 (from 137.4 ± 9.8 to 135.4 ± 10.0; *p* < 0.001) ↑ 0.95% EC4 (from 137.4 ± 9.8 to 138.7 ± 11.4; *p* < 0.05) EC1, EC2 and EC3 increase performance compared to EC4 (*p* = 0.001)

CAFF: Caffeine; Cr: Creatine; EC: Experimental condition; EG: Experimental group; min: Minute; s: second; SB: Sodium bicarbonate; *: Statistical differences (*p* < 0.05) in the comparison of an intervention based on the intake of a sport supplement compared to a placebo.

### 3.4. Quality assessment and risk of publication bias

A total of 23 studies were included, with 16 studies (69.6%) rated as excellent and 7 studies (30.4%) rated as good according to the PEDro scale. The mean score of the studies is 9.04. Therefore, the quality of the studies included in this systematic review can be qualified as excellent.

According to the author’s criteria, the funnel plots (SMD against standard error) were notably symmetrical, indicating an absence of publication bias. Funnel plots from different comparisons are presented on Supplementary File 1.

### 3.5. Risk of bias

The studies demonstrated a low risk of bias in Domains 1, 2, 3, and 4, while Domain 5 was categorized as “some concerns” due to the absence of a protocol in the included studies, except for two [4,48]. Table 3 summarizes the risk of bias of the included studies.

**Table 3.** Quality assessment of the studies included in the systematic review.

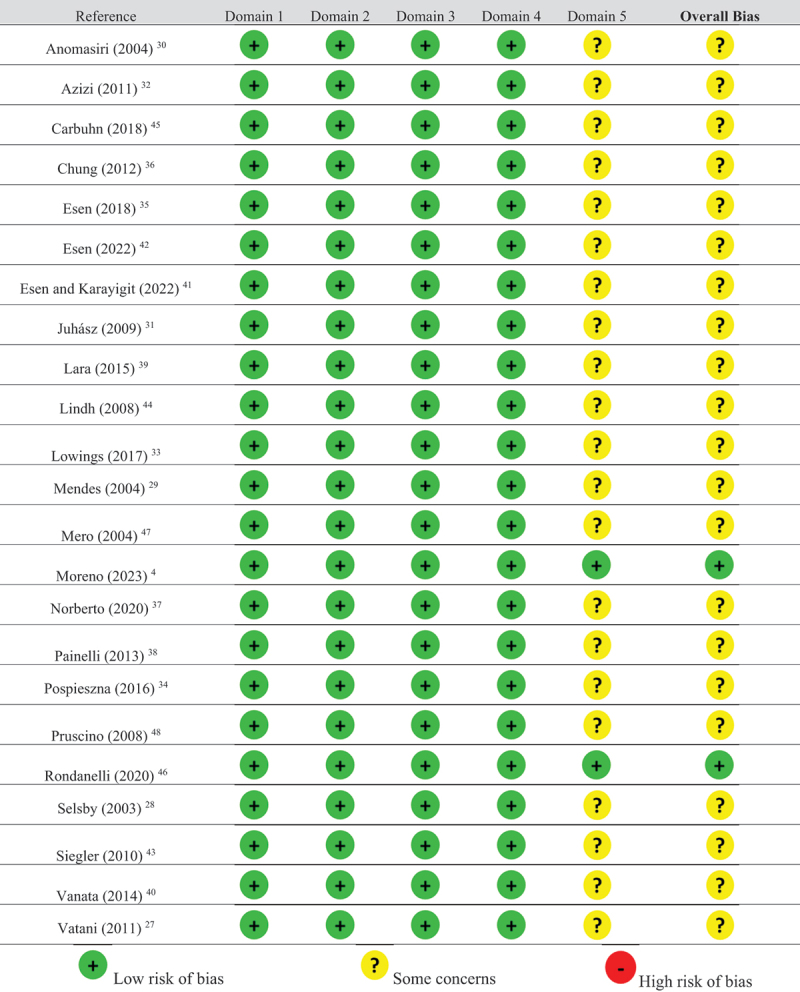

### 3.6. Meta-analyses

**Figure 2.**
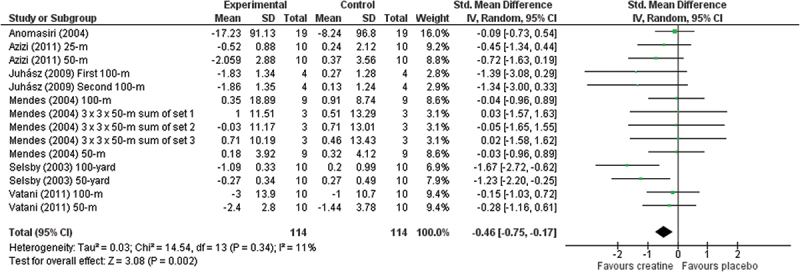
Forest plot illustrating intervention-related (creatine supplementation) changes in performance in comparison to placebo. Forest plot values are shown as effect sizes with 95% confidence intervals. Green squares: Individual studies and size represents relative weight. Black rhomboid: summary value.

**Figure 3.**
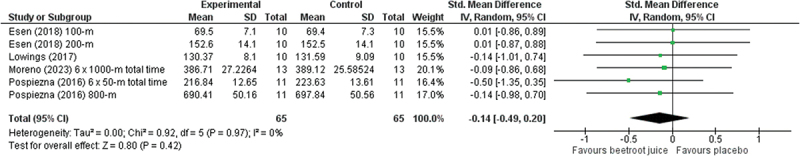
Forest plot illustrating intervention-related (beetroot juice) changes in performance in comparison to placebo. Forest plot values are shown as effect sizes with 95% confidence intervals. Green squares: Individual studies and size represents relative weight. Black rhomboid: Summary value.

**Figure 4.**
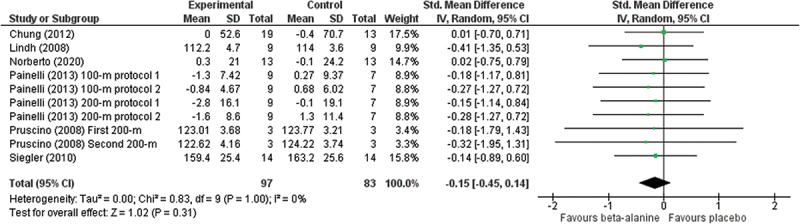
Forest plot illustrating intervention-related (beta-alanine and sodium bicarbonate) changes in performance in comparison to placebo. Forest plot values are shown as effect sizes with 95% confidence intervals. Green squares: individual studies and size represents relative weight. Black rhomboid: Summary value.

**Figure 5.**
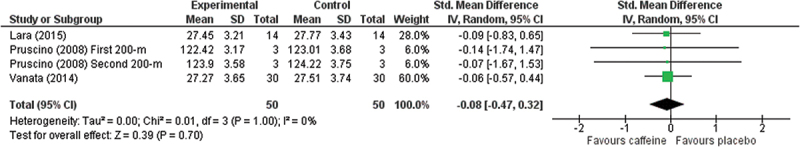
Forest plot illustrating intervention-related (caffeine supplementation) changes in performance in comparison to placebo. Forest plot values are shown as effect sizes with 95% confidence intervals. Green squares: Individual studies and size represents relative weight. Black rhomboid: summary value.

Meta-analysis reports a significant effect of Cr (ES = −0.46; 95% CIs = −0.75 to −0.17, *p* = 0.002; *I*^2^ = 11%) on performance and improving it compared to placebo (Figure 2), while the other analyzed supplements as BRJ (ES = −0.14; 95% CIs = −0.49 to 0.20, *p* = 0.42; *I*^2^ = 0%), Beta-alanine and SB (ES = −0.15; 95% CIs = −0.45 to 0.14, *p* = 0.31; *I*^2^ = 0%) and caffeine (ES = −0.08; 95% CIs = −0.47 to 0.32, *p* = 0.70; *I*^2^ = 0%) showed a non-significant improvements in performance when compared to placebo (see Figure 3–5, respectively). Meta-analyses of L-arginine/L-citrulline, Probiotic (B. longum 35,624) and rice germ were not possible based on the aforementioned criteria.

### 3.7. Certainty of evidence

Certainty of evidence based on GRADE model is shown in Table 4.

**Table 4.** Risk of bias assessment based on RoB 2 scale.

Supplement	Number of included studies (number of participants)	Certainty of evidence	Downgraded reason
Creatine	6 (*228*)	Moderate	Imprecision
Beetroot juice	4 (*130*)	Moderate	Imprecision
β-alanine and sodium bicarbonate	6 (*180*)	Moderate	Imprecision
Caffeine	3 (*100*)	Moderate	Imprecision
L-arginine/L-citrulline	2	Very low	Insufficient number of studies to perform meta-analysis
Probiotic (B. longum 35,624)	1	Very low	Insufficient number of studies to perform meta-analysis
Rice germ	1	Very low	Insufficient number of studies to perform meta-analysis

### 3.7. Sensitivity analyses

Sensitivity analyses showed no substantial changes when omitting each single study in meta-analyses, excepting for creatine supplementation, which *p*-value converted into *p* = 0.06 when omitting the study conducted by Selsby et al. [30].

## 4. Discussion

Based on the studies included in this review, only the following SS were eligible for meta-analysis: Cr, BRJ, caffeine, β-alanine, and SB. The meta-analysis revealed that Cr had a significant positive effect on swimming performance (ES = −0.46, *p* = 0.002), while the other SS did not demonstrate any significant impact.

### 4.1. Effects of creatine supplementation

Cr supplementation, compared to placebo, has demonstrated ergogenic effects based on the results of the meta-analysis. Cr is among the SS with the highest prevalence of use in the athletic population [51], with a prevalence of 28.3% between competitive swimmers [13]. Cr is a nitrogen-containing compound classified within the family of guanidine phosphagens. It is synthesized endogenously from methionine, arginine, and glycine in the kidneys [52]. Endogenous synthesis supplies only half of the daily Cr requirements (~2 g/day) [53]. Cr supplementation has been associated with improved explosive and high-intensity efforts because in all-out efforts around 1–6 s intramuscular adenosine triphosphate (ATP) and phosphocreatine (PCr) are the main metabolic pathways [54]. The breakdown of PCr into phosphate and Cr by creatine kinase (CK) releases approximately 10.3 kcal, which can be utilized to resynthesize adenosine diphosphate (ADP) and phosphate into ATP [52]. Given the limited availability of PCr, increasing Cr and PCr levels through supplementation offers a potential mechanism to mitigate fatigue and improve performance and recovery [55].

Attending to the potential mechanism of Cr for increasing swimming performance, Mendes et al. [31] reported in 50-m, 100-m and an interval protocol (3 × 3 x 50-m) a faster time in all the test used, however mean time did not reach statistical significance (*p* > 0.05). In contrast, Cr increased lean body mass (LBM). Considering that structural hypertrophy requires a minimum of 8–12 weeks of resistance training [56], Cr supplementation could lead to nonfunctional hypertrophy due to increased water accumulation at the intramuscular level, driven by the high osmotic power of Cr [57]. Consequently, the potential metabolic benefits might be limited by its indirect effects on body composition. In this way, Azizi [34] reported that Cr supplementation enhanced vertical jump ability and bench press performance during a high-intensity test but failed to improve swimming performance in either the 25-m or 50-m events among female competitive swimmers.

Selsby et al. [30] reported an improvement in 100-yard performance and a greater reduction in time compared to a placebo in both 50-yard and 100-yard events following supplementation. Similarly, Vatani et al. [29] observed a 4.5% faster 50-m time in trained male swimmers. Additionally, a study conducted with international adolescent swimmers [32] reported a reduced time to complete two sets of 100 m, interspersed with 4-min rest periods. Furthermore, in a longer distance (400 m), Cr supplementation resulted in a higher velocity during the final 50 m of the test.

The results of this systematic review and meta-analyses suggest that this supplement increases swimming performance, especially during interval procedures [32], although Cr could induce an increase in water content [29–31,33]. These effects could be mediated by an increased muscle Cr and PCr concentrations that could increase to resynthesize ATP [52]. However, the studies included in this review had a limited duration (ranging from 6 to 14 days). Future research should investigate the potential for greater adaptations to swimming training and the adaptive responses to resistance exercise programs integrated into swimming training regimens [58]. Therefore, it is important to minimize potential excess water retention as a response to Cr supplementation. Additionally, it should be noted that swimmers with low dietary Cr intake (e.g. vegetarians) are more sensitive to the ergogenic effects of this supplement [55].

### 4.2. Effects of beetroot juice supplementation

BRJ has not been shown to significantly enhance swimming performance based on the currently available literature. However, some swimmers may benefit from its intake. BRJ is a dietary source rich in inorganic nitrate (NO₃^−^), which is partially reduced (~20%) to nitrite (NO₂^−^) by anaerobic facultative bacteria located on the dorsal surface of the tongue. Nitrite is further reduced to nitric oxide (NO) in the stomach [59], under hypoxic conditions [60], and in acidotic environments [61]. The physiological effects of NO, such as enhancing type II muscle fiber contraction, reducing the ATP cost of force production, and improving muscle blood flow, could contribute to ergogenic benefits [62]. Nevertheless, the prevalence of the intake of BRJ between competitive swimmers is scarce [13]. In this systematic review, four included studies analyzed the effect of BRJ in swimmers. The results of the meta-analysis reported a non-significant improvement of this supplement in swimming performance.

Esen et al. [37] found no differences in performance during either a 100-m or a 200-m test following BRJ ingestion compared to a placebo. In contrast, Moreno et al. [4] observed a trend toward statistical differences in the final sets of an interval protocol consisting of 6 × 100 m, although the differences did not reach statistical significance. Using a higher dose of NO₃^−^ (12.5 mmol vs. the 6.5 mmol used by Esen et al. [37] and Moreno et al. [4]), Lowings et al. [35] reported a trend toward improved performance in a 168-m test, particularly during the second half of the trial (*p* = 0.06). Similarly, with a high dose of NO₃^−^ (10.2 mmol), Pospieszna et al. [36] reported faster performance in the final four sets of an interval protocol consisting of 6 × 50 m. Considering that the ergogenic effect of BRJ is mediated by its capacity of increasing NO respect to the baseline values [63], and a threshold for detecting a statistical response to supplementation upper 8.4 mmol NO_3_^−^ [64], it is possible that the amount of NO_3_^−^ used in the studies of Moreno et al. [4] and Esen et al. [37] could be insufficient for detecting statistical differences. Therefore, based on the results of this systematic review, the potential ergogenic effect of BRJ appears to be limited to doses exceeding 8.4 mmol of NO₃^−^ consumed approximately 3 h prior to exercise. However, these possible ergogenic properties should be confirmed by additional studies.

### 4.3. Effects of β-alanine and sodium bicarbonate supplementation

The systematic review with meta-analysis included an evaluation of β-alanine and SB supplementation, focusing specifically on high-intensity efforts lasting between 6 and 60 s. These efforts are associated with the generation of hydrogen ions (H^+^), which contribute to a reduction in intramuscular pH [54]. A decline in pH can trigger a cascade of physiological responses, including the inhibition of phosphofructokinase [6], which impairs the glycolytic pathway, disrupts phosphocreatine recovery, and affects muscle contraction by altering the competition between calcium (Ca^2 +^) and H^+^ at the troponin-binding site [65]. Additionally, it influences the release and reuptake of Ca^2 +^ within the sarcoplasmic reticulum [66]. Moreover, a decrease in pH can elevate the rate of perceived exertion (RPE) [67]. One of the interventions analyzed in this review is β-alanine, a limiting factor in the synthesis of carnosine within muscle tissue [68]. Carnosine acts as a calcium transporter within the sarcoplasmic reticulum [69], facilitating increased cross-bridge formation and potentially enhancing muscle contraction speed. Additionally, it plays a key role in maintaining intracellular acid-based balance, thereby regulating intramuscular pH and supporting sustained ATP production through non-oxidative metabolism [69].

Several studies investigated the effects of β-alanine supplementation on swimmers. Painelli et al. [40] reported significant enhancements in 100-m and 200-m time trial tests after a six-week period of β-alanine supplementation, but Chung et al. [38] did not find significant differences between β-alanine and placebo in their study. Although no improvements were observed in a 400-m time trial test among national-level swimmers in another study [39], the selected distance and its greater reliance on glycolytic metabolism in such longer events may have reduced sensitivity to the potential ergogenic effects of β-alanine supplementation. In addition, the potential ergogenic benefits of β-alanine supplementation may be more pronounced in vegetarians or swimmers with limited intake of animal-based dietary sources, due to their lower baseline carnosine levels [70]. To mitigate the side effect of paresthesia, which occurs in a dose-dependent manner, it is recommended to gradually increase β-alanine dosages from 0.8–1.6 g/day to 4.8–6.4 g/day [70,71].

SB plays a critical role in maintaining blood pH levels [72]. During high-intensity swimming, the body can become increasingly acidotic. In such efforts, the elevated HCO₃^−^ levels following SB ingestion enhance the buffering capacity of excess H^+^ in the blood. This helps regulate intramuscular pH and delays the accumulation of H^+^ within muscle fibers. The overall effect is an increased reliance on the glycolytic pathway during high-intensity efforts and a postponement of fatigue onset [73].

Some studies examining SB supplementation in swimmers have shown promising results, despite the non-significant effects observed in the meta-analysis. Elite male swimmers demonstrated improved performance in a 200-m time trial test [46], aligning with performance enhancements reported during interval training sessions in non-elite university swimmers [45]. These findings align with the established effectiveness of SB in other sports, such as endurance events, high-intensity exercises, and high-intensity intermittent efforts [74].

Nonetheless, SB supplementation is associated with side effects such as abdominal pain, nausea, vomiting, and bloating, which can vary in severity depending on the dose. To optimize performance while minimizing side effects, it is essential to adhere to recommended dosages. The suggested protocol involves ingesting 300 mg/kg of SB approximately 180 min prior to exercise, preferably in enteric-coated capsules and alongside a carbohydrate-rich meal, with dosages tailored to individual responses [75,76].

### 4.4. Effects of caffeine supplementation

Caffeine (1,3,7 trimethylxanthine) is the SS most consumed by elite swimmers [13]. Caffeine is one of the SS with the strongest scientific evidence supporting its ergogenic properties, which are mediated through the blocking of adenosine receptors [77]. At central level, caffeine affect to arousal [78] and mood [79] for optimizing predisposition to exercise. In addition, dopamine synthesis is increased [80]. The increased neuro excitability and central effect contribute to the effectiveness of caffeine for reducing RPE and pain [81]. After caffeine, spinal neurons become more excited [82], facilitating increased muscle recruitment [83]. In addition, caffeine increases the activity of the Na_2_^+^/K^+^ potassium [84], and Ca_2_^+^ bioavailability in the muscle [85]. In fact, caffeine has been shown to increase movement velocity across the full range of submaximal loads in resistance exercise [86]. It also enhances power output at the same level of electromyographic activity, which can be considered an indicator of neuromuscular efficiency [87], and enables greater work output at the same RPE [88].

In swimming, CAFF enhanced the time to cover a 50-m test in the habitual swimming style in national level swimmers [41] and in a 50-yard test among competitive swimmers [42]. However, the results of our meta-analysis did not reveal a significant effect of caffeine compared to placebo. It is worth noting that only three studies were included in meta-analysis, which may have influenced the outcome. Therefore, further research is needed to better understand the effects of CAFF on swimmers.

### 4.5. Effects of other sport supplements

Supplements without strong level of scientific evidence, such as L-arginine/L-citrulline, probiotic and rice germ [12], have been studied as possible ergogenic supplements in competitive swimmers. L-arginine/L-citrulline are precursors of NO. NO is synthesized through a nitric oxide synthase (NOS)-dependent pathway from L-arginine and oxygen in a reaction catalyzed by enzymes such as endothelial nitric oxide synthase (eNOS). Additionally, L-citrulline can serve as a precursor for NO synthesis through its conversion to L-arginine [89]. Physiological effects of L-arginine and L-citrulline are mediated by its capacity of increasing NO. These effects include increased muscle blood flow, cardiac output, gas exchange, and enhanced bioavailability of oxidative nutrients [90], and to increased muscle contraction and delayed muscular fatigue [91]. However, one study analyzing the effects of L-arginine supplementation in a 200-m and L-arginine and L-citrulline supplementation in a 100-m time trial test failled for detecting any effects in swimming performance [44]. Therefore, the results of this systematic review align with previous findings suggesting that alternative NO precursors, such as arginine and L-citrulline, have an unclear effect on enhancing endurance performance [89].

The gut microbiota contains numerous bacterial species with physiological effects linked to various diseases and general health parameters. Carbuhn et al. [47] investigated the effects of a specific probiotic strain, *Bifidobacterium longum* 35624, during an intensive training program in university-level female swimmers. Although the study did not report any significant effects of this supplement on 100-m or 500-m time trial performance, a slightly improved perception of recovery was observed during the final 2 weeks of the 6-week intervention. However, as this is the only study available on this topic, this systematic review suggests conducting further research to explore the potential effects of *Bifidobacterium longum* 35624 on recovery and training adaptations in response to intensive swimming training programs.

Rice germ has gained attention as a potential candidate for nutraceutical and pharmaceutical research due to its rich nutrient composition [92]. One study reported that consuming 25 g of rice germ twice daily for 5 weeks led to improved performance, demonstrated by faster 200-m swim times [48]. However, these findings should be interpreted with caution, as the study did not account for potential confounding factors related to energy or macronutrient intake, nor did it use a placebo with comparable energy and macronutrient content. Furthermore, the mechanisms underlying the observed performance improvements were not explained by the authors.

### 4.6. Effects of the co-ingestion of more than a supplement

Athletes who consume sports supplements often take more than one SS simultaneously [93]. In fact, 86.9% of competitive swimmers report using multiple SS [13], with an average consumption of 5.5 ± 3.5 supplements. The effects of co-ingesting different SS with ergogenic properties are not simply additive, and the impact of combined supplementation is not yet fully understood [93].

β-alanine helps regulate intramuscular pH by promoting the efflux of H^+^ to the extracellular space via carnosine-mediated transport [94], while SB facilitates H^+^ efflux to the blood through a concentration gradient (a noncompetitive mechanism with β-alanine) and enhances glycolytic energy contribution. Given the absence of competition between these mechanisms, Painelli et al. [40] investigated the combined effects of SB and β-alanine supplementation versus a placebo over 5 weeks in trained swimmers, focusing on 100-m and 200-m time trials. The study found improved performance in the 100-m test only among participants who combined β-alanine and SB. For the 200-m test, faster times were observed with β-alanine alone, SB alone, and their co-ingestion. Although no statistical differences were detected between β-alanine and SB alone versus their co-ingestion, the combined supplementation showed the greatest average reduction in 200-m time (co-ingestion: 2.13%; β-alanine alone: 1.22%; SB alone: 1.46%). Based on these findings, the combined intake of β-alanine and SB may be suggested as an ergogenic strategy for competitive swimmers.

The positive effects of SB, mediated by its ability to regulate both extracellular [7] and intramuscular pH during high-intensity efforts [73], appear unrelated to the potential enhancement of the phosphagen system following Cr ingestion [55]. To investigate a possible positive interaction between SB and Cr, Mero et al. [49] analyzed the effects of a 6-day Cr supplementation protocol combined with acute SB supplementation during a 2-repetition 100-m interval procedure. The study reported a reduction in the incremental time to complete the second 100-m compared to the first under placebo conditions. The authors suggested that the recovery time in the study may have been insufficient to allow complete restoration of PCr stores [49]. Therefore, the stimulation of PCr resynthesis from Cr supplementation, combined with faster restoration mediated by enhanced pH regulation, could potentially increase PCr turnover, contributing to the improvements observed with SB and Cr co-ingestion. The implications of this study suggest that co-ingestion of SB and Cr could enhance interval training sessions, potentially leading to greater training adaptations. However, the absence of groups receiving only Cr or SB makes it necessary to interpret these findings with caution and await further studies to confirm these results.

The acid-base regulation mediated by SB [70] may be enhanced by both the central [78–80] and peripheral effects [82–84] of caffeine. To investigate a potential synergistic effect of combining caffeine and SB, Pruscino et al. [50] examined the co-ingestion of CAFF and SB during two 200-m sets. The study found that CAFF improved performance in the first 200-m compared to a placebo; however, the magnitude of the improvement was observed with the combined effect of CAFF+SB or SB alone.

The authors suggested that while caffeine may enhance performance in a single time-trial test, it could impair recovery [50]. The absence of performance differences in the second 200-m may indicate a potential benefit in interval protocols during the initial efforts, as maintaining the same performance at the end could be interpreted as an indicator of similar fatigue levels. This study is the only one that did not report an ergogenic effect of SB supplementation.

### 4.7. Strengths, limitations and future perspectives

SS consumption is a frequent practice between competitive swimmers of both sexes [13–15] who are qualified as polyconsumers. Considering that the effects of SS on sports performance depend on the metabolic and mechanical characteristics of each sport modality [53], and that recommendations should be grounded in scientific evidence derived from meta-analyses [12], this study may serve as a guide for planning SS selection for swimmers based on robust scientific findings.

It is important to highlight that the present study has several limitations. Firstly, analysis of publication bias was performed through visual inspection of funnel plots. In this line, the extended Egger’s test [95] could allow us to quantify this analysis, but unfortunately this test is usually performed only if ≥10 studies per outcome were available and the studies of the present study did not meet this criteria. In addition, meta-regression could allow us to determine if any of the intervention variables (i.e. dose of the supplement, distance assessed or participant characteristics) predicted the effects of supplementation on swimmers performance, but this analysis is usually performed only if ≥10 studies per outcome were available [96]. This fact demonstrates that further research on the topic could help to establish firmer conclusions and the optimal dose of SS to enhance performance. In addition, there is a limited number of studies that included female participants, and most of them did not report results stratified by sex. This limitation in analyzing mixed-sex samples prevents the assessment of sex-specific interactions with the ergogenic properties of SS. Given the underrepresentation of female participants in studies investigating the effects of SS on sports performance [97], as well as the lack of precise sex-specific results, it is imperative for future research to focus on examining the ergogenic properties of SS with consideration for the sex of the participants. It is important to note that our sensitivity analyses showed a change from significant findings (i.e. *p* < 0.05) to non-significant findings when omitting the study conducted by Selsby (2003). However, even omitting this study, the p-value showed was *p* = 0.06, which shows a clear trend to signification. This fact, together with the high methodological quality (Table 5) and the absence of a high risk of bias (Table 4) showed by this study means that the conclusions of our study should not be altered based on the findings of our sensitivity analysis. However, it is important to highlight the potential effect this study may have on the findings presented in this systematic review with meta-analysis to interpret them with caution. Finally, despite the moderate certainty of evidence showed in meta-analyses in the present study, practitioners should apply the results of the present systematic review with meta-analysis with caution, since limited evidence is currently available to establish firm conclusions with recommended dosages.

**Table 5.** Quality assessment of the studies included in the systematic review.

Reference	Item 1	Item 2	Item 3	Item 4	Item 5	Item 6	Item 7	Item 8	Item 9	Item 10	Item 11	Points	Methodological quality
Anomasiri [30]	Yes	Yes	Yes	Yes	Yes	Yes	Yes	Yes	Yes	Yes	Yes	10	Excellent
Azizi [32]	Yes	Yes	Yes	Yes	Yes	Yes	Yes	Yes	Yes	Yes	Yes	10	Excellent
Carbuhn [45]	Yes	Yes	Yes	Yes	Yes	Yes	Yes	Yes	Yes	Yes	Yes	10	Excellent
Chung [36]	No	Yes	Yes	Yes	Yes	Yes	Yes	No	Yes	Yes	Yes	9	Excellent
Esen [35]	Yes	Yes	Yes	Yes	Yes	Yes	Yes	Yes	Yes	Yes	Yes	10	Excellent
Esen [42]	Yes	Yes	Yes	Yes	Yes	Yes	Yes	Yes	Yes	Yes	Yes	10	Excellent
Esen and Karayigit [41]	Yes	Yes	Yes	Yes	Yes	Yes	Yes	No	Yes	Yes	Yes	9	Excellent
Juhász [31]	Yes	Yes	Yes	Yes	Yes	Yes	Yes	Yes	Yes	Yes	Yes	10	Excellent
Lara [39]	Yes	Yes	Yes	Yes	Yes	Yes	Yes	Yes	Yes	Yes	Yes	10	Excellent
Lindh [44]	Yes	Yes	Yes	Yes	Yes	Yes	Yes	Yes	Yes	Yes	Yes	10	Excellent
Lowings [33]	Yes	Yes	Yes	Yes	Yes	Yes	Yes	No	Yes	Yes	Yes	9	Excellent
Mendes [29]	No	Yes	Yes	Yes	Yes	Yes	Yes	Yes	Yes	Yes	Yes	10	Excellent
Mero [47]	Yes	No	Yes	Yes	Yes	Yes	Yes	Yes	No	No	No	6	Good
Moreno [4]	Yes	Yes	Yes	Yes	Yes	Yes	Yes	Yes	No	No	No	7	Good
Norberto [37]	Yes	Yes	Yes	Yes	Yes	No	Yes	No	Yes	Yes	Yes	8	Good
Painelli [38]	Yes	Yes	Yes	Yes	Yes	Yes	Yes	Yes	Yes	Yes	Yes	10	Excellent
Pospieszna [34]	Yes	Yes	Yes	Yes	Yes	Yes	Yes	Yes	No	No	No	7	Good
Pruscino [48]	Yes	Yes	Yes	Yes	Yes	Yes	Yes	Yes	Yes	Yes	Yes	10	Excellent
Rondanelli [46]	Yes	Yes	Yes	Yes	No	No	No	Yes	Yes	Yes	Yes	7	Good
Selsby [28]	Yes	Yes	Yes	Yes	Yes	Yes	Yes	Yes	Yes	Yes	Yes	10	Excellent
Siegler [43]	Yes	Yes	Yes	Yes	Yes	No	No	Yes	Yes	Yes	Yes	8	Good
Vanata [40]	Yes	Yes	Yes	Yes	Yes	No	No	Yes	Yes	Yes	Yes	8	Good
Vatani [27]	Yes	Yes	Yes	Yes	Yes	Yes	Yes	Yes	Yes	Yes	Yes	10	Excellent

## 5. Conclusions

The present systematic review and meta-analysis on SS in swimming provided valuable insights into their potential effects on performance. Cr supplementation emerged as a promising option, demonstrating performance improvements, particularly in shorter-distance events such as the 100 m and 200 m. BRJ, despite its known ability to increase NO levels, did not significantly enhance swimming performance according to the meta-analysis. While the meta-analytic results for β-alanine and SB were inconclusive, individual studies suggested potential benefits, particularly in contexts such as shorter distances and high-intensity efforts. Caffeine, one of the most widely consumed supplements among athletes, did not demonstrate significant effects in the meta-analysis. Nonetheless, individual studies indicated potential performance benefits, highlighting the need for further research in swimming-specific contexts. One intriguing finding was the potential benefits of combining specific supplements. The co-ingestion of β-alanine and SB showed promise for enhancing swimming performance, particularly in interval training sessions. Similarly, combining Cr and SB may have a positive impact on adaptations to interval training. In conclusion, this systematic review underscores the potential advantages of select SS in improving swimming performance. However, supplementation strategies should be personalized to meet individual needs and training demands, with the guidance of qualified professionals. Professional oversight and further research are essential to optimizing supplementation strategies for competitive swimmers.

## Data Availability

The datasets used and/or analyzed during the current study are available from the corresponding author on reasonable request.
